# Möbius Syndrome With Possible Poland Syndrome Overlap: A Case Report

**DOI:** 10.7759/cureus.79916

**Published:** 2025-03-02

**Authors:** Richa Agarwal, Manish Kumar, Abhimanyu Vasudeva, Palani Selvam Mohanraj, Shivani Jain

**Affiliations:** 1 Ophthalmology, All India Institute of Medical Sciences, Gorakhpur, IND; 2 Pediatrics, All India Institute of Medical Sciences, Gorakhpur, IND; 3 Physical Medicine and Rehabilitation (PMR), All India Institute of Medical Sciences, Gorakhpur, IND; 4 Biochemistry, All India Institute of Medical Sciences, Gorakhpur, IND; 5 General Practice, Jain Hospital, Gorakhpur, IND

**Keywords:** abducens nerve, eye movements, facial asymmetry, pectoralis muscles, poland syndrome

## Abstract

Möbius syndrome (MBS) is a rare congenital neurological disorder. It is primarily characterized by underdevelopment of the facial and abducens nerves, leading to facial paralysis and limited eye movement. Musculoskeletal anomalies and neurodevelopmental challenges add to its complexity.

Neuroimaging, particularly magnetic resonance imaging (MRI), often shows brainstem hypoplasia and flattening of the fourth ventricle floor.

This case report describes a six-year-old child with facial asymmetry, incomplete eye closure, and abnormal head posture. These features suggest facial and abducens nerve palsy, both key signs of Möbius syndrome. The child also had pectoral muscle atrophy and nipple deformity, raising the possibility of Poland syndrome. This condition has occasionally been reported alongside Möbius syndrome. Clinical evaluations were consistent with these findings. Genetic testing was not performed but could provide further insights.

The overlap between Möbius and Poland syndrome highlights the complexity of these disorders. A multidisciplinary approach is essential for management, and an early and thorough clinical evaluation is key to timely diagnosis and intervention.

## Introduction

Möbius syndrome (MBS) is a rare congenital neurological disorder primarily marked by underdevelopment of the facial (cranial nerve VII) and abducens (cranial nerve VI) nerves, leading to facial paralysis and limited eye movement. The condition is also associated with musculoskeletal abnormalities, dental anomalies, and, in some instances, neurodevelopmental impairments. Due to its rarity, comprehensive clinical descriptions remain limited, making it a challenging disorder to identify [1].

Recent advances in neuroimaging have provided deeper insights into the underlying pathology of MBS. Magnetic resonance imaging (MRI) findings in affected individuals commonly reveal hypoplasia of the brainstem, often characterized by flattening of the fourth ventricle floor due to the absence of the facial colliculus. These imaging abnormalities correlate with both clinical symptoms and neurophysiological assessments, highlighting their importance in diagnosing the disorder [2].

A study of 37 Dutch patients further delineated the syndrome, with 97% exhibiting bilateral ocular abduction weakness, 48% conjugate horizontal gaze paresis, and 34% features of Duane retraction syndrome, in addition to motor impairments, lingual dysfunction, and respiratory abnormalities. These findings suggest that Möbius syndrome extends beyond a cranial nerve disorder, instead representing a broader rhombencephalic maldevelopment affecting motor nuclei, axonal pathways, and long tracts [3].

A unified pathogenic hypothesis links Möbius syndrome with Poland anomaly, Klippel-Feil anomaly, Sprengel deformity, and terminal transverse limb defects, proposing that vascular compromise of the subclavian and vertebral arteries during the sixth week of gestation underlies these congenital conditions. Further research is needed to elucidate the mechanisms driving embryonic vascular disruption and its role in congenital malformation syndromes [4].

Poland syndrome presents with underdevelopment or absence of the breast, nipple, and subcutaneous tissue, along with partial or complete absence of the pectoralis major and minor muscles. It may also involve missing costal cartilages or ribs (commonly 2-4 or 3-5). A chest wall defect, sometimes accompanied by lung herniation, is a frequent feature. However, clinical expression varies widely, and all characteristics are rarely seen in a single individual [5].

Klippel-Feil syndrome, defined by congenital fusion of cervical vertebrae, is frequently accompanied by other anomalies like Sprengel deformity and scoliosis, suggesting its involvement in a wider spectrum of congenital disorders [6].

Möbius sequence is a congenital disorder characterized by bilateral facial nerve palsy and external ophthalmoplegia, often associated with limb and orofacial anomalies. While its precise etiology remains debated, growing evidence suggests that vascular disruption during early embryogenesis, specifically within the subclavian artery supply disruption sequence (SASDS), plays a critical role. A case report describes an infant with bilateral facial paralysis (cranial nerve VII), external ophthalmoplegia (cranial nerves IV and VI), and involvement of cranial nerves V, IX, X, XI, and XII, alongside Poland anomaly, terminal transverse limb defects, and diaphragmatic agenesis. Neuroimaging and histopathology revealed brainstem calcifications and diffuse brainstem injury, suggesting vascular insufficiency affecting the proximal sixth intersegmental artery before the sixth week of gestation [7].

Möbius syndrome (MBS) is a congenital cranial dysinnervation disorder (CCDD) characterized by bilateral palsy of the abducens (CN VI) and facial (CN VII) nerves, often accompanied by other cranial nerve deficits, particularly those in the dorsal pons and medulla oblongata. It is a rare disorder, occurring in 1:50,000 to 1:500,000 live births, with no gender predilection. Three primary etiological theories have been proposed: vascular disruption, genetic mutations affecting facial motor nucleus development, and teratogenic exposure (e.g., misoprostol) during early pregnancy. While these theories have traditionally been considered independently, recent research aims to integrate them at a molecular level. Due to its low prevalence and diagnostic complexity, MBS may be underdiagnosed, highlighting the need for further investigation into its pathogenesis and molecular mechanisms [8].

## Case presentation

**Figure 1:**
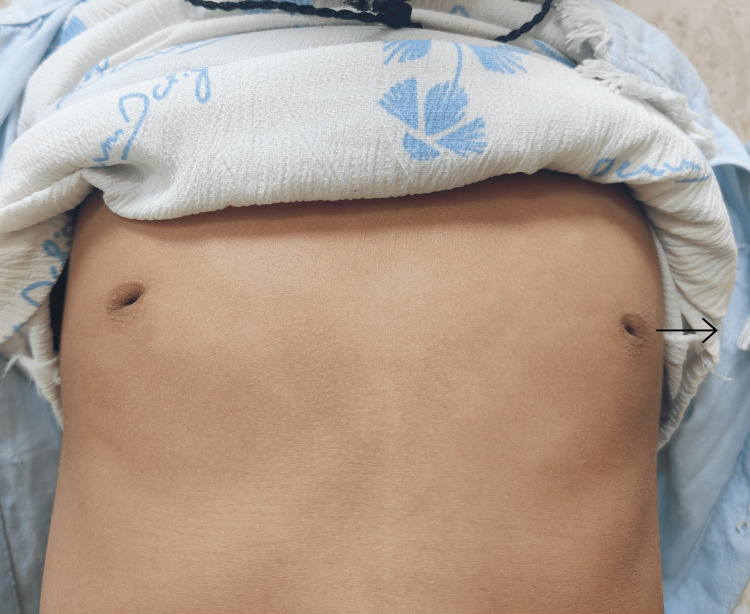
Image showing deformed nipples

**Figure 2:**
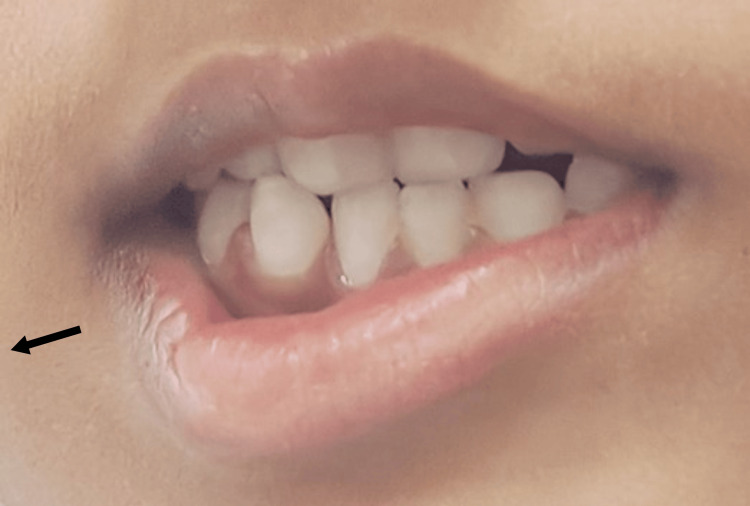
Left-sided facial nerve palsy (During attempted smiling, deviation of the corner of the mouth toward the right was observed, indicative of left-sided facial nerve palsy. The arrow denotes the direction of deviation.)

A six-year-old patient presented at a tertiary care hospital in North India with complaints of facial asymmetry, and deviation of the face to the right side during emotional expressions (e.g., laughing and crying). The child had an uneventful birth history. There was no reported family history of similar craniofacial abnormalities or related conditions. Upon further examination, it was noted that the patient had deformed nipples (Figure 1), pectoralis muscle atrophy, facial asymmetry and incomplete eye closure on the left side, raising concerns of facial nerve involvement. Consequently, the case was further investigated for facial nerve palsy. The ENT examination revealed incomplete eyelid closure on the left side and facial asymmetry, which was more pronounced during smiling and crying. The facial nerve palsy was suspected to be Grade 3 according to the House-Brackmann scale for facial paralysis [9] on the left side (Figure 2).

**Figure 3:**
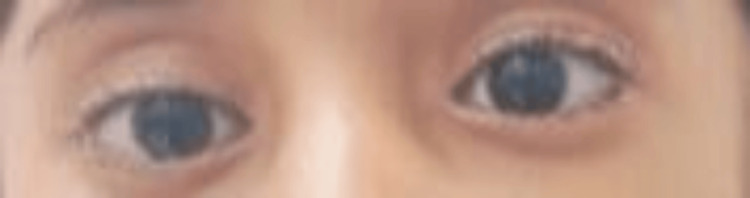
Right eye shows esotropia of 20 PD (The PBCT measurement of 20 PD in primary gaze applies to both distance and near.)

**Figure 4:**
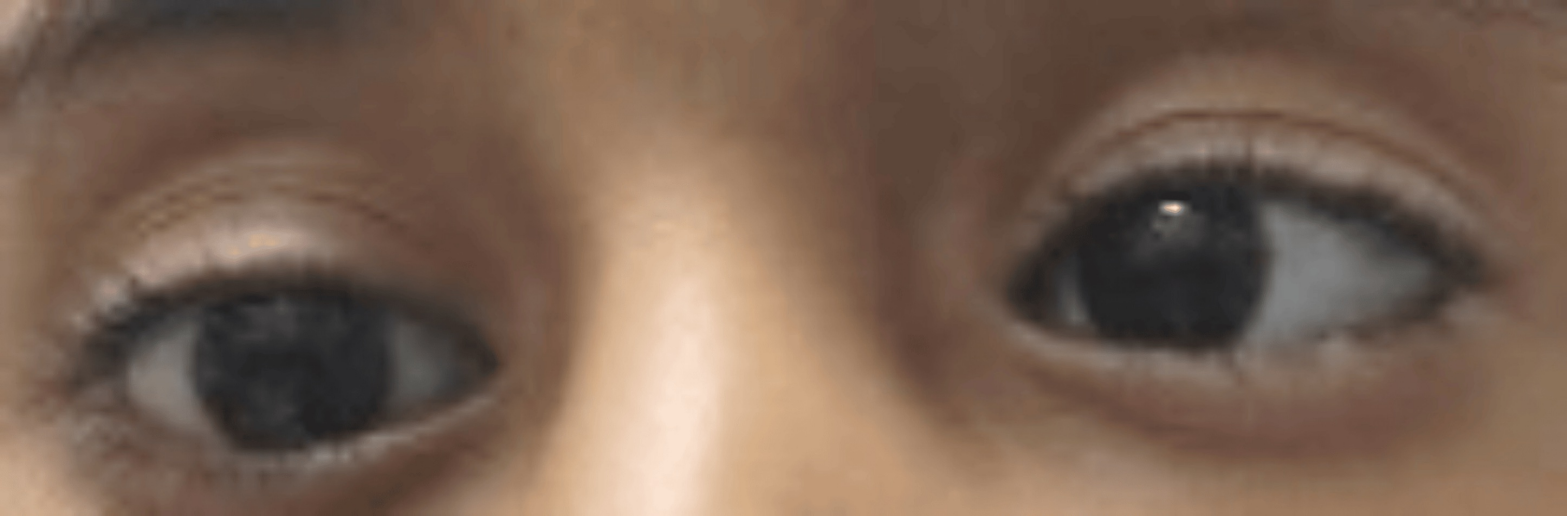
Right eye shows limited abduction on attempted dextroversion.

The diagnosis of Möbius syndrome is established after excluding other conditions with similar presentations. One important differential is Melkersson-Rosenthal syndrome, which is characterized by a triad of congenital facial palsy, tongue fissuring, and lip swelling. Poland syndrome is another relevant differential, as it can present with congenital facial and abducens nerve palsies accompanied by ipsilateral underdevelopment of the pectoralis muscle. Other conditions that must be ruled out include hereditary congenital facial palsy (HCFP), which involves isolated facial palsy without abducens nerve involvement; Duane retraction syndrome, marked by restricted horizontal eye movement, globe retraction, and palpebral fissure narrowing; congenital fibrosis of the extraocular muscles, which manifests as severe congenital strabismus, ptosis, and vertical gaze palsy; as well as DiGeorge syndrome and CHARGE syndrome, both of which have broader multisystem involvement [10]. Further investigations were recommended to confirm the diagnosis. Audiological evaluation of the patient indicated normal hearing in the right ear, as confirmed by Pure Tone Audiometry (PTA), while moderate conductive hearing loss (CHL) was identified in the left ear. Otoacoustic emissions (OAE) testing yielded a passing result for the right ear, whereas results for the left ear were inconclusive, necessitating further assessment. Brainstem Evoked Response Audiometry (BERA) demonstrated a replicable Wave V at 65 dB HL in the left ear, consistent with moderate hearing loss, while the right ear exhibited Wave V at 35 dB HL, indicating normal auditory function. These findings suggest unilateral conductive hearing loss in the left ear, with normal hearing in the right ear. The ophthalmological examination revealed a best-corrected visual acuity of 20/40 in the right eye and 20/20 in the left eye, with no significant refractive error. The patient exhibited left-sided facial nerve palsy with inadequate closure of the left eye. She also had an abnormal head posture, characterized by a face turn to the right, which is likely compensatory for ocular misalignment. There was 20PD of esotropia in the right eye. While a light reflex would have been beneficial for assessment, it could not be obtained due to the child's limited cooperation. While a light reflex would have been beneficial for assessment, it was not performed due to the child's limited cooperation. Extraocular movements were limited in the right eye, with decreased abduction (Figure 3 and Figure 4).

The left eye demonstrated normal extraocular movement. A forced duction test (FDT) was done, which was free for the lateral rectus (LR), but positive for the medial rectus (MR) of the right eye, suggesting possible MR contracture, while the FDT for the left eye was free for both the MR and LR muscles. The presence of MR contracture in the right eye is indicative of a longstanding LR palsy rather than an acute-onset palsy, supporting a congenital etiology in this young child. The absence of restriction in the left eye suggests normal muscle function.

Additionally, the forced generation test was negative for the lateral rectus of the right eye, indicating paralysis of LR. The anterior segment examination and fundus were both within normal limits, with no pathological findings observed. Based on these, the patient was diagnosed with right-eye strabismic amblyopia, right-sided 6th nerve palsy (abducens nerve palsy), and left-sided facial nerve palsy. These findings are consistent with the clinical presentation of Moebius syndrome. The definition and diagnostic criteria of Moebius syndrome have been subject to ongoing debate and challenges [11]. The contralateral involvement of the abducens and facial nerves represents a novel phenotype that could contribute to a deeper understanding of the condition. Based on our search, this specific presentation has not been previously reported.

A cardiac evaluation through echocardiography revealed normal findings. An MRI of the brain and orbit showed no significant abnormalities, and an ultrasound of the abdomen and pelvis, performed to assess for visceral anomalies, also yielded normal results. The overall clinical findings raised suspicion for a syndromic presentation pointing towards Moebius syndrome, which overlapped with Poland syndrome.

The patient was initiated on occlusion therapy for amblyopia, with patching of the left eye for 4-6 hours per day. Monthly follow-ups were scheduled to assess treatment response and determine the need for strabismus surgery. For lagophthalmos, lubricating eye drops were prescribed during the day, along with a lubricating gel at bedtime. Nighttime patching was not advised, as Bell’s phenomenon was intact with no corneal exposure. Additionally, a structured facial rehabilitation program was implemented, incorporating massage therapy, blinking and eye closure exercises, lip and cheek strengthening, blowing exercises, and mirror therapy, with regular follow-ups to monitor progress and adjust treatment as needed.

Since both syndromes impact the patient's physical appearance, their management strategies share common principles. Treatment for Möbius syndrome primarily involves surgical interventions to enhance facial movement, while Poland syndrome often necessitates chest wall reconstruction to improve function and reduce associated morbidity. In cases where breast development is affected, gender-specific reconstructive procedures such as mammoplasty may be required. A long-term approach may also be necessary, involving staged surgeries or rehabilitation to optimize outcomes. The overarching goal in both conditions is to restore symmetry and enhance the patient’s overall quality of life [12]. The potential genetic contribution of these syndromes was taken into account during family counseling.

Written informed consent was obtained from the patient’s legal guardian after providing a detailed explanation of the purpose, procedures, potential benefits, and possible risks associated with the evaluation and publication of clinical findings. All questions were addressed, and consent was given voluntarily in accordance with ethical guidelines and institutional protocols.

## Discussion

A study of 40 individuals with congenital facial weakness (aged 23 months to 64 years) identified four distinct ocular motor patterns, with the most common (43%) being bilateral horizontal gaze palsy with intact vertical range. Other deficits included vertical saccadic dysfunction (34%), impaired vertical optokinetic responses (68%), and reduced convergence (66%), suggesting broader involvement of midbrain ocular motor structures beyond pontine abducens nuclei dysfunction. This classification of ocular motor deficits may provide insights into anatomic localization and underlying genetic mechanisms in MBS and related disorders [13].

A study reported that while the majority of individuals diagnosed with Möbius syndrome exhibit its typical features, approximately one in five present with vertical gaze limitations or do not fully meet the established diagnostic criteria. Careful assessment of ocular motility may help in refining classification, which could contribute to a better understanding of prognosis, inform genetic evaluation, and support further research into the condition’s underlying causes [14].

In another article, Poland-Möbius syndrome - a rare condition marked by cranial nerve VI and VII dysfunction, pectoral muscle hypoplasia, and limb anomalies - remains a subject of debate regarding whether it represents a distinct disorder or a phenotypic variation within a broader developmental spectrum. Genetic and environmental factors are believed to play a role, with PLXND1 (3q21-q22) implicated in neurogenesis and vasculogenesis. It also presents the first documented case of Poland-Möbius syndrome linked to a maternally inherited PLXND1 mutation (NM_015103.2:ex14:c.2890G>A, p.V964M), suggesting its potential involvement in Möbius syndrome. A review of 14 additional cases with genetic data found no further evidence implicating PLXND1, though prior research has associated it with isolated Möbius syndrome. These findings support the possibility of a genetic overlap between Möbius and Poland syndromes, further implicating PLXND1 in their shared developmental origins [15].

There is about a 15% association between Moebius syndrome and Poland syndrome [16]. This overlapping phenotype, or Moebius-Poland syndrome, is a rare congenital disorder with an estimated prevalence of 1 in 500,000 and is characterized by cranial nerve VI and VII palsies along with pectoral muscle dysplasia, among other features [17]. The putative explanation for this overlap is that both Poland syndrome and Moebius syndrome result from an interruption of early embryonic blood supply in the subclavian or vertebral arteries and/or their branches, with prenatal exposure to teratogens being a potential contributing factor. These two syndromes are often grouped together under the umbrella term subclavian artery supply disruption sequence (SASDS) [16].

Given the likely shared embryonic origin of Moebius syndrome and Poland syndrome, as outlined above, the primary distinction between the two lies in their characteristic features: cranial nerve VI and VII palsies in Moebius syndrome and absence or hypoplasia of the pectoral muscles in Poland syndrome. Although not exhaustive, the clinical features used to describe Moebius and Poland syndromes are presented in the table (Table 1).

**Table 1:** Distinguishing Between Möbius and Poland Syndromes

Moebius Syndrome [10,18]	Poland Syndrome [19]
Cranial nerve abnormalities: CN VI and/or VII (partial or complete) or other cranial nerve involvement.	Agenesis or hypoplasia of the pectoralis major muscle (cardinal feature). Usually unilateral but bilateral involvement has been reported.
Lower extremity abnormalities: Talipes equinovarus, syndactyly, ankylosis, absent phalanges, longitudinal or transverse deficits	Agenesis or hypoplasia of other chest muscles
Upper extremity abnormalities: Digital hypoplasia, ectrodactyly	Thoracic deformities such as pectus carinatum/excavatum, clavicular hypoplasia, or pulmonary herniation
Facial abnormalities: Cleft palate, micrognathia, microtia, microphthalmia	Mammary abnormalities such as hypoplastic breast, areola, nipple
Thoracic abnormalities: Pectoral hypoplasia, breast anomaly, chest wall deformity	Other abnormalities such as Sprengel anomaly, asymmetric axillary hairs, radioulnar synostosis, or vertebral anomalies

## Conclusions

This case highlights the necessity of a multidisciplinary approach in diagnosing and managing rare congenital syndromes such as Möbius and Poland syndromes. A thorough evaluation is essential for early diagnosis, allowing for timely and individualized management. In this patient, ongoing care should include routine follow-ups in Ophthalmology, Otolaryngology, and Physical Medicine and Rehabilitation to address facial nerve palsy, ocular motility limitations, and associated developmental concerns.

The observed phenotype - left-sided facial palsy, right-sided abducens palsy, and unilateral conductive hearing loss in the left ear - represents a unique phenotype that may enhance our understanding of the condition. To the best of our knowledge, this specific presentation has not been documented before.

## References

[1] Monawwer SA, Ali S, Naeem R (2023). Moebius syndrome: an updated review of literature. Child Neurol Open.

[2] Pedraza S, Gámez J, Rovira A, Zamora A, Grive E, Raguer N, Ruscalleda J (2000). MRI findings in Möbius syndrome: correlation with clinical features. Neurology.

[3] Verzijl HT, van der Zwaag B, Cruysberg JR, Padberg GW (2003). Möbius syndrome redefined: a syndrome of rhombencephalic maldevelopment. Neurology.

[4] Bavinck JN, Weaver DD, Opitz JM, Reynolds JF (1986). Subclavian artery supply disruption sequence: hypothesis of a vascular etiology for Poland, Klippel-Feil, and Möbius anomalies. Am J Med Genet.

[5] Urschel HC Jr (2009). Poland syndrome. Semin Thorac Cardiovasc Surg.

[6] Menger RP, Rayi A, Notarianni C (2021). Klippel-Feil syndrome. In: StatPearls [Internet].

[7] St Charles S, DiMario FJ Jr, Grunnet ML (1993). Möbius sequence: further in vivo support for the subclavian artery supply disruption sequence. Am J Med Genet.

[8] López Gutierrez D, Luna López I, Medina Mata BA, Moreno Castro S, García Rangel FY (2024). Physiopathologic bases of Moebius syndrome: combining genetic, vascular, and teratogenic theories. Pediatr Neurol.

[9] Mat Lazim N, Ismail H, Abdul Halim S, Nik Othman NA, Haron A (2023). Comparison of 3 grading systems (House-Brackmann, Sunnybrook, Sydney) for the assessment of facial nerve paralysis and prediction of neural recovery. Medeni Med J.

[10] Zaidi SM, Syed IN, Tahir U, Noor T, Choudhry MS (2023). Moebius syndrome: what we know so far. Cureus.

[11] (2025). Moebius syndrome; MBS [Internet]. https://omim.org/entry/157900.

[12] Chopan M, Sayadi L, Laub D (2015). Mobius syndrome and Poland syndrome presenting together in a single patient. Eplasty.

[13] Rucker JC, Webb BD, Frempong T, Gaspar H, Naidich TP, Jabs EW (2014). Characterization of ocular motor deficits in congenital facial weakness: Moebius and related syndromes. Brain.

[14] MacKinnon S, Oystreck DT, Andrews C, Chan WM, Hunter DG, Engle EC (2014). Diagnostic distinctions and genetic analysis of patients diagnosed with moebius syndrome. Ophthalmology.

[15] Glass GE, Mohammedali S, Sivakumar B, Stotland MA, Abdulkader F, Prosser DO, Love DR (2022). Poland-Möbius syndrome: a case report implicating a novel mutation of the PLXND1 gene and literature review. BMC Pediatr.

[16] Bissonnette B, Luginbuehl I, Engelhardt T (2019). Poland-Moebius syndrome. In: Syndromes: Rapid Recognition and Perioperative Implications.

[17] Dubrey SW, Patel MC, Malik O (2009). Moebius-Poland syndrome and drug associations. BMJ Case Rep.

[18] Abramson DL, Cohen MM Jr, Mulliken JB (1998). Möbius syndrome: classification and grading system. Plast Reconstr Surg.

[19] Baldelli I, Baccarani A, Barone C (2020). Consensus based recommendations for diagnosis and medical management of Poland syndrome (sequence). Orphanet J Rare Dis.

